# PCIS1 is an essential factor in mitochondrial RNA splicing and complex I biogenesis, with distinct effects in null and downregulated mutants

**DOI:** 10.1007/s00299-026-03821-w

**Published:** 2026-06-29

**Authors:** Ella Kobaivanov, Brody Frink, Mizuki Takenaka, Oren Ostersetzer-Biran

**Affiliations:** 1 Department of Plant and Environmental Sciences, The Alexander Silberman Institute of Life Sciences, The Hebrew University of Jerusalem, Jerusalem, Israel; 2 Department of Botany, Graduate School of Science, Kyoto University, Sakyo-Ku, Kyoto, 606-8502 Japan; 3 Institute of Parasitology, Biology Centre, Czech Academy of Sciences, České Budějovice, Czech Republic

**Keywords:** PCIS1 (PPR co-expressed intron splicing-1), Group II intron, Splicing, Mitochondria, Land plants, Arabidopsis thaliana

## Abstract

**Key message:**

PCIS1 is indispensable for plant mitochondria NAD4 and NAD7 gene expression. Null mutants abolish CI assembly and arrest embryogenesis, whereas knockouts retain partial splicing, revealing a genetic functional mt-RNA metabolism threshold.

**Abstract:**

Plant mitochondrial biogenesis relies on the splicing of numerous group II introns, primarily in genes encoding complex I (CI) subunits, which require multiple nuclear-encoded cofactors. Recently, we characterized PPR co-expressed intron splicing-1 (PCIS1), an essential factor identified in silico, based on its co-expression with several pentatricopeptide repeat (PPR) proteins. Knockout of Arabidopsis PCIS1 results in embryonic arrest, but mutant lines can be maintained via embryo-specific *ABI3*-driven expression. The partially complemented *pABI3::PCIS1* plants show growth and developmental defect phenotypes, associated with defective processing of *nad2*, *nad4*, and *nad7* mitochondrial transcripts. To eliminate the potential leakiness of tissue-specific promoters and better define PCIS1 function, we analyzed the RNA and protein profiles in homozygous *pcis1* seedlings generated by a modified embryo-rescue approach. Our data confirm that PCIS1 is essential for respiratory biogenesis via splicing of *nad4* intron 3 and *nad7* introns 1 and 2, and that null and downregulated *pcis1* lines display distinct phenotypic and molecular defects. Analysis of different *pcis1* mutant lines links the embryogenesis defects to impaired CI assembly and respiratory dysfunction, and reveals distinct consequences of PCIS1 knockdown versus null mutations for mt-RNA processing, OXPHOS biogenesis and plant physiology.

**Supplementary Information:**

The online version contains supplementary material available at 10.1007/s00299-026-03821-w.

## Introduction

Mitochondria contain their own genomes (mtDNAs, or mitogenomes), with intrinsic machineries to support transcription, RNA processing and translation that are required for the expression of the set of organellar genes, including structural RNAs (i.e., tRNAs and rRNAs) and proteins required in the oxidative phosphorylation (OXPHOS) activities (Bonen 2018; Gualberto and Newton 2017; Knoop 2012). During evolution, mitochondria lost many ancestral genes and became increasingly dependent on nuclear-encoded factors for organellar biogenesis and respiratory functions. In addition to OXPHOS-related proteins, these include numerous nuclear factors which evolved to support organellar gene expression (Fuchs, et al. 2020). The respiratory system consists of four major electron transport chain (ETC) complexes (CI-CIV) and the ATP synthase enzyme (CV), which are embedded in the inner mitochondrial membrane (Braun 2020; Møller, et al. 2021). In addition to the canonical ETC enzymes, plant mitochondria also harbor several alternative oxidases (AOXs) and alternative NAD(P)H dehydrogenases (NDs) proteins, which can bypass parts of the electron transport chain and likely provide metabolic flexibility under stress or fluctuating respiratory conditions (Møller, et al. 2021).

Although mitochondria share a common bacterial origin, they have diversified considerably across eukaryotic lineages. Plant mitogenomes are notably large and structurally complex, encoding more genes than animal mitochondrial genomes (Bonen 2018; Gualberto and Newton 2017; Knoop 2012). A distinctive feature of plant mitochondria is the extensive RNA processing required for maturation of primary transcripts (Best, et al. 2020a, b; Hammani and Giege 2014; Small, et al. 2023; Zmudjak and Ostersetzer-Biran 2018). These include RNA editing (Small, et al. 2020) and the splicing of group II-type introns that reside within many essential organellar proteins (Bonen 2008; Brown, et al. 2014). Canonical group II introns are autocatalytic RNAs assisted by intron-encoded maturases for splicing in vivo (Lambowitz and Belfort 2015; Lambowitz and Zimmerly 2011; Mizrahi, et al. 2022; Schmitz-Linneweber, et al. 2015; Zhao and Pyle 2017a, b; Zimmerly and Semper 2015). In plant mitochondria, however, the introns have diverged in structural features and lost the coding genes for their cognate maturases. Instead, their splicing relies on nuclear-encoded RNA-binding cofactors, such as maturase-related proteins, RNA helicases, and plant-specific splicing cofactors (Best, et al. 2020a, b; Hammani and Giege 2014; Small, et al. 2023; Zmudjak and Ostersetzer-Biran 2018). Among these, pentatricopeptide repeat (PPR) proteins represent a major family of organellar RNA-binding factors (Small and Peeters 2000). These contain tandem helix-turn-helix repeats that confer sequence-specific RNA recognition (Barkan, et al. 2012; Gully, et al. 2015; Yagi, et al. 2013). While the role of PPR proteins in RNA editing is well established, their specific functions in group II intron splicing remain largely unclear. PPR proteins may assist intron folding and most likely function together, directly or indirectly, with additional cofactors to facilitate splicing.

Here, we report the analysis of the Pentatricopeptide Repeat Co-expressed Intron Splicing-1 (PCIS1) factor, encoded by the *AT5G25500* locus in *Arabidopsis thaliana* plants. PCIS1, originally identified based on its co-expression with PPR proteins, is proposed to function as a candidate maturation factor of several *nad* transcripts in Arabidopsis mitochondria (Frink, et al. 2024). Similar to other nuclear-encoded proteins essential for mitochondrial gene expression, loss of PCIS1 causes early embryonic arrest. Knockdown mutant lines can be maintained through embryo-specific expression of PCIS1 driven by the *ABI3* promoter (Despres, et al. 2001; Frink, et al. 2024; Nakashima, et al. 2006). The *pABI3::PCIS1* lines exhibited reduced germination and impaired growth, associated with respiratory defects due to defective processing of *nad4*, *nad7* and *nad2* transcripts. As gene expression driven by tissue-specific promoters is leaky (Ng, et al. 2014), and to better define the role of PCIS1 in mitochondrial (mt-) RNA metabolism, we generated homozygous *pcis1* lines using a modified embryo-rescue approach (Best, et al. 2023; Shevtsov-Tal, et al. 2021). The analysis of homozygous plants established the roles of PCIS1 in the splicing of *nad4* intron 3 and *nad7* intron 2, whereas the altered *nad2* RNA processing, previously reported for the *pABI3::PCIS1* lines, likely represents a secondary effect that was also observed in various other mitochondrial RNA-processing mutants; see e.g., (Best, et al. 2023; Colas des Francs-Small and Small 2014). Our results highlight key differences between PCIS1 downregulation and null mutations in mitochondrial RNA processing, OXPHOS biogenesis, and plant physiology. The effects of PCIS1 knockout on RNA splicing and the differences between homozygous knockout and *ABI3*-driven knockdown lines in organellar biogenesis and the physiology of embryo-rescued Arabidopsis mutants are described in detail.

## Materials and methods

### Plant material and growth conditions

*Arabidopsis thaliana* (Col-0) wild type and mutant-lines (i.e., *pcis1-1*, *pcis-1–2*) were obtained from the ABRC at Ohio State University (Columbus, OH). The construction of the partially (hemi-) complemented *ABI3::PCIS1* line is described in Frink, et al. (2024). Before germination, seeds were surface-sterilized with a sodium hypochlorite (NaOCl) solution and sown on MS agar plates supplemented with 1% (w/v) sucrose. Petri plates were stratified in the dark at 4°C for 5 days, then transferred to a controlled growth chamber (22°C, 100 µE·m⁻^2^·s⁻^1^) under short-day (8 h light/16 h dark) or long-day (16 h light/8 h dark) conditions. PCR screening was performed to verify the genotype of each line using specific oligonucleotides (Table S1). Sequencing of PCR products confirmed the precise T-DNA insertion sites.

### Embryo-rescue and establishment of homozygous mutants

Embryo rescue of the *pcis1-1* mutant line (Frink, et al. 2024) was carried out following the method described in (Best, et al. 2023; Shevtsov-Tal, et al. 2021). Siliques from Col-0 and heterozygous *pcis1-1* plants, collected about 10 days after anthesis, were surface-sterilized using 6% sodium hypochlorite (NaOCl) solution for 10 min at room temperature (RT), followed by a 10 min incubation in 70% ethanol solution at RT. The siliques were then rinsed with sterile double-distilled water (DDW). Subsequently, they were carefully opened in a biological hood under a binocular microscope for the extraction of the green and white seeds, which then were plated on Murashige and Skoog (MS) agar medium supplemented with 3% (w/v) sucrose and vitamins (i.e., 100 µg myoinositol, 1.0 µg thiamine, 1.0 µg pyridoxine, 1.0 µg nicotinic acid per liter of media). To generate sufficient biomass for RNA and protein extraction, seedlings at the six-leaves (L6) stage (Boyes, et al. 2001) were transferred to liquid MS-medium containing 1–3% sucrose and vitamins (Shevtsov-Tal, et al. 2021), and cultivated under the growth conditions outlined in the ‘Plant material and growth conditions’ section, with gentle agitation (~ 50 to 100 RPM).

### Analysis of the RNA profiles of wild type (col-0) and *pcis1* plants

RNA was extracted from 50 mg of tissue of Arabidopsis wild type plants or mutant-lines using Tri-Reagent (Sigma, St. Louis, MO, USA), essentially as described previously (Best, et al. 2023; Cohen, et al. 2014; Shevtsov-Tal, et al. 2021). The RNA was treated with DNase I (RNase-free) prior to its use in the RT-qPCR reactions, with oligonucleotides designed for specific different exon–exon (for the analysis of processed transcripts) or intron–exon regions (for the analysis of primary pre-RNAs) in Arabidopsis mitochondria (Table S2). *GAPDH* (At1g13440), *ACTIN2* (At3g1878), *18S-rRNA* (At3g41768), *RRN26* (Atmg00020), *RRN5* (Atmg01380) and *RRN18* (Atmg01390) genes were used as reference genes.

### Analysis of the protein profiles in wild type and *pcis1* mutant plants

Crude membrane extracts were prepared as previously described (Pineau, et al. 2008). Whole seedlings of wild type (Col-0) and *pcis1* mutant plants were homogenized in 2 ml of extraction buffer (75 mM MOPS, pH 7.6; 0.6 M sucrose; 4 mM EDTA; 0.2% PVP-40; 8 mM L-cysteine; 0.2% BSA). The lysate was filtered through Miracloth and centrifuged at 1300 × g to remove debris. Crude organellar membrane fractions were collected by centrifugation at 22,000 × g for 10 min at 4°C. Proteins were solubilized in either native (for blue-native, BN) or denaturing loading buffer and separated by PAGE analysis. After electrophoresis, proteins were transferred to PVDF membranes and incubated overnight at 4°C with specific primary antibodies (Table S3), followed by detection using chemiluminescence after incubation with HRP-conjugated secondary antibodies.

### Analysis of native organellar complexes by BN-PAGE

Blue-native (BN) PAGE was performed as previously described (Colas des Francs-Small, et al. 2012; Eubel, et al. 2005; Pineau, et al. 2008). Membrane fractions, corresponding to 200 mg of plant tissue from wild type and mutant lines, were solubilized in 1.5% (w/v) n-dodecyl-*β*-maltoside (DDM) using ACA buffer (750 mM aminocaproic acid, 0.5 mM EDTA, 50 mM Tris–HCl, pH 7.0) and incubated on ice for 30 min. After centrifugation at 20,000 × g for 10 min, the supernatant was mixed with 0.2% (v/v) Serva Blue G and loaded onto a 4.5–16% native gradient gel. Proteins were transferred to PVDF membranes using cathode buffer (50 mM Tricine, 15 mM Bis–Tris-HCl, pH 7.0) at 40 mA for 15 h at 4°C. Immunodetection was carried out with specific primary antibodies and HRP-conjugated secondary antibodies, followed by chemiluminescence detection.

### Respiratory activity in Arabidopsis wild type and mutant-lines

Oxygen consumption was measured at 22°C in darkness using a Clark-type oxygen electrode (Hansatech Instruments, Norfolk, UK), with data acquisition performed using Oxygraph Plus v1.01 software, following the protocol of Tomaz, et al. (2010). The electrode was calibrated by depleting dissolved O₂ with excess sodium dithionite in ~ 1.0 ml of autoclaved tap water. Whole plants were cut into small pieces with scissors, and the tissues were pre-incubated in the dark for 15 min in a buffered solution (10 mM HEPES, 10 mM MES, 2.0 mM CaCl₂, pH 7.2). Total respiration rates were then recorded following the addition of 25 mg of tissue to 2.0 ml of autoclaved tap water.

## Results

### The *PCIS1* gene (AT5G25500) encodes a conserved mitochondrial protein required for embryogenesis and early plant establishment

**Fig. 1:**
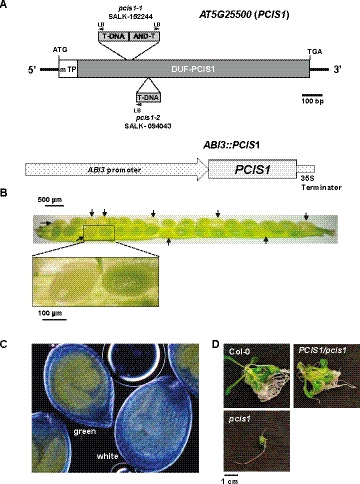
The *Arabidopsis PCIS1* (*AT5G25500*) gene encodes an essential mitochondria-localized protein. **A** Schematic representation of the PCIS1 (AT5G25500) gene locus. The boxes represent different gene regions, including an N-terminal region with homology to a mitochondrial localization sequence (Emanuelsson, et al. 2000) and a conserved domain in flowering plants of unknown (unassigned) function, i.e., DUF-PCIS1. The locations of the T-DNA insertion sites are indicated. The lower panel shows the *ABI::PCIS1* gene construct, containing the PCIS1 coding region under the regulation of the Arabidopsis ABI3 (*AT3G24650*) promoter region (Despres, et al. 2001; Frink, et al. 2024; Nakashima, et al. 2006), and the 35S transcription terminator site. **B** Siliques at the green-mature stage obtained from heterozygous *pcis1-1* plants. **C** Green (wild type or heterozygous) and translucent/pale-green seeds containing homozygous *pcis1-1* embryos were analyzed by differential interference contrast (Nomarski) microscopy. **D** Seeds at the late torpedo stage of wild type (Col-0) plants, heterozygous (*PCIS1/pcis1*) and homozygous *pcis1* mutant plants were germinated and grown in vitro on MS-agar plates supplemented with 3% sucrose. The image shows 10-week-old rescued seedlings grown at 22°C, 50% RH, under long-day conditions (18 h light/6 h dark) with a light intensity of ~ 100 µE·m⁻^2^·s⁻^1^. Genotypes were confirmed by PCR and sequencing. Bars represent sizes as indicated in each panel

The genome of *A. thaliana* encodes a well-conserved gene in angiosperms termed as ‘*PPR Co-expressed Intron Splicing-1*’ (*PCIS1*, *AT5G25500*) (Fig. 1A). PCIS1 was originally characterized based on its co-expression pattern with several PPR proteins (Frink, et al. 2024). The *PCIS1* gene locus encodes a 420-amino acid protein (UniProt: Q5XV34_ARATH) with an N-terminal mitochondrial targeting pre-sequence (Frink, et al. 2024; Hooper, et al. 2017), followed by a conserved region of unknown function in flowering plants, annotated here as ‘DUF-PCIS1’ (Fig. 1A). The InterPro (www.ebi.ac.uk/interpro/) server currently (as of September 2025) annotates this region as “EXOSOME COMPLEX EXONUCLEASE” (PANTHER PTHR37763:SF1) (Blum, et al. 2024). However, there are no experimental or structural evidence available to support this annotation. Moreover, previous studies showed that PCIS1 influences the processing and maturation of several mitochondrial transcripts (i.e., *nad2*, *nad4*, and *nad7* pre-RNAs) in Arabidopsis plants (Frink, et al. 2024). ‘The Arabidopsis Information Resource’ (TAIR) database indicates that *PCIS1* is a lowly expressed gene, whose product appears to accumulate at higher levels in early developing tissues, such as during early germination, in apical meristems, young leaves, and floral tissues (Fig. S1). Although the functions of PCIS1 are critical during embryogenesis, knockdown mutants with reduced *PCIS1* expression were established through temporal gene suppression under an embryo-specific (i.e., *ABI3*) promoter (Frink, et al. 2024). Yet, gene expression driven by tissue-specific promoters, including ABI3 (Ng, et al. 2014), is often considered leaky; caution is required when interpreting the resulting growth and developmental phenotypes, as well as the RNA and protein data, corresponding to the *ABI3::PCIS1* line. To better investigate the role(s) of PCIS1 in organellar RNA processing, we generated and analyzed homozygous *pcis1* mutant lines by a modified embryo-rescue method (Best, et al. 2023; Shevtsov-Tal, et al. 2021). These included the SALK_152244 (*pcis1-1*) and SALK_094043 (*pcis1-2*) lines, both carrying T-DNA insertions within the coding region of the *PCIS1* gene locus (Fig. 1A). Sequencing of genomic PCR products spanning the gene, T-DNA insertion junctions confirmed the integrity of the wild type and mutant lines.

As previously noted by Frink, et al. (2024), heterozygous *pcis1-1* and *pcis1-2* plants produced siliques in which approximately one-quarter of the seeds (39/156 and 117/463, respectively) were deformed, displaying a translucent morphology (Fig. 1B, 1C), and later developed into shrunken or wrinkled seeds (Figs. S2 A, S2B), a seed morphology previously associated with delayed embryogenesis and impaired cellular energy metabolism (Focks and Benning 1998; Kristof, et al. 2008). In contrast, Arabidopsis wild type (Col-0) plants produced fewer than 0.5% abortive seeds (1/208). The ‘normal’ and ‘*wrinkled*’ seeds were germinated on MS plates. While normal seeds displayed nearly complete germination, the wrinkled/abnormal seeds exhibited markedly reduced germination rates, with fewer than 25% of seeds initiating germination. Many of these abnormal seedlings (homozygous *pcis1* mutants) died shortly after germination on MS-medium, but a small number of plants were able to establish and propagate on soil (Fig. S2 C). In Arabidopsis, certain mitochondrial mutants, including *pcis1* mutants (Fig. S2 C), exhibit accumulation of anthocyanin and ‘curly leaf’ phenotype that is often accompanied by growth defects (Colas des Francs-Small and Small 2014). Such phenotypes, arising from impaired mitochondrial function, can impact multiple developmental processes (e.g., through alterations in auxin-related signaling pathways) (Christensen, et al. 2000), including leaf morphology, root formation, and seed development (Figs. S2 C, S2D). In *pcis1* homozygous mutants, some plants were able to produce a small number of non-viable seeds (Fig. S2 D, left panel), whereas others exhibited severe defects in embryo and seed development, leading to complete seed abortion (Fig. S2 D, right panel). These results indicate that loss of PCIS1 function causes embryonic developmental defects, consistent with PCIS1 being essential for normal embryo development, likely due to disrupted mitochondrial RNA metabolism during embryogenesis.

### Establishing homozygous Arabidopsis *pcis1* mutant lines

Similar to various Arabidopsis mutants affected during embryogenesis, including those impaired in mitochondrial biogenesis (Cohen, et al. 2014; Cordoba, et al. 2016; Dahan, et al. 2014; Fromm, et al. 2016), the white *pcis1-1* seeds failed to germinate on soil (i.e., under in vivo conditions), or died shortly after radicle emergence. Differential interference contrast (DIC, Nomarski) microscopy analysis indicated that the development of *pcis1* embryos is arrested at the torpedo stage (Figs. 1C, S2). One effective strategy for investigating the post-embryonic functions of essential genes is partial genetic complementation, in which the gene of interest is expressed under a seed- or embryo-specific promoter, such as *ABI3* (Despres, et al. 2001) or *RPS5A* (Johnson, et al. 2008). This approach has successfully facilitated the study of embryo-arrested mutants (Aryamanesh, et al. 2017; Sun, et al. 2018), including those affected in organellar RNA metabolism, and has also proven suitable for characterizing *pcis1* mutants (Frink, et al. 2024).

Here, we tested the germination of *pcis1-1* seeds under in vitro conditions previously shown to support the establishment of homozygous Arabidopsis mutants affected in mt-RNA metabolism. Some embryo-arrested mutants of Arabidopsis can germinate on Murashige and Skoog (MS) plates supplemented with high (1–3%) sugar concentrations, or, in some cases, through more elaborate embryo-rescue methods in vitro (Best, et al. 2023; Dahan, et al. 2014; Shevtsov-Tal, et al. 2021). Embryo rescue of the *pcis1-1* (SALK_152244) and *pcis1-2* (SALK_094043C) mutant lines was carried out following a modified embryo-rescue method described previously (Best, et al. 2023; Shevtsov-Tal, et al. 2021). For this purpose, green (wild type or heterozygous) and white (homozygous) seeds were obtained from green mature surface-sterilized siliques and plated on MS agar medium supplemented with 3% (w/v) sucrose and vitamins (see Material and Methods). Under these conditions, a small number of *pcis1-1* and *pcis1-2* seeds were able to germinate on MS agar medium supplemented with sucrose (, and S2). To generate sufficient biomass for RNA and protein analyses, seedlings at the six-leaves (L6) stage (Boyes, et al. 2001) were transferred into liquid MS-medium containing 1–3% sucrose and vitamins (Shevtsov-Tal, et al. 2021). PCR-based genotyping confirmed that the resulting seedlings were indeed homozygous for the SALK_152244 T-DNA insertion. As controls, we compared the organellar RNA and protein profiles, respiratory function, and physiology of wild type and heterozygous plants obtained from germinated embryos of the same progeny (i.e., green seeds) at the same developmental stage (i.e., late torpedo stage), as well as partially (*hemi*-) complemented *ABI3::PCIS1* lines (Frink, et al. 2024). The rescued homozygous seedlings exhibited slow growth, requiring 3 ~ 4 months to reach the ‘L6’ stage (Boyes, et al. 2001), and progressed beyond this stage only after being transferred to a liquid MS-based rescue medium; see also (Best, et al. 2023; Shevtsov-Tal, et al. 2021). For the analysis of RNA and protein profiles, as well as organellar activities, we used six-month-old embryo-rescued *pcis1-1* and *pcis1-2* seedlings. In parallel, wild type (Col-0) plants and *ABI3::PCIS1* seedlings at the equivalent developmental stage (L6), were analyzed in comparable molecular and biochemical assays.

### PCIS1 plays a key role in the processing of *nad4* and *nad7* transcripts in Arabidopsis mitochondria

**Fig. 2:**
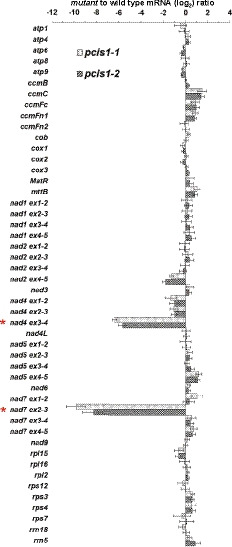
Altered abundance of mitochondrial *nad4* and *nad7* RNA transcripts in *pcis1* mutant plants. Total RNA extracted from whole seedlings of wild type (Col-0) plants and *pcis1-1* or *pcis1-2* mutant lines was analyzed by reverse transcription followed by quantitative real-time PCR (RT-qPCR). Transcript levels were normalized to multiple internal references, including *GAPDH* (*AT1G13440*), *ACTIN2* (*AT3G18780*), *18S rRNA* (*AT3G41768*), and *rrn26* (mitochondrial 26S rRNA, *ATMG00020*). RT-qPCR was performed to assess the expression profiles of mRNAs corresponding to the 32 protein-coding genes in Arabidopsis mitochondria. The histograms show the log2 ratio of the relative RNA levels in *pcis1* lines compared with those of the wild type plants. Data represent the mean values of four biological replicates, each with 12 technical repeats; error bars indicate one standard deviation

In *Arabidopsis thaliana* mitochondria, the coding regions of various genes encoding CI subunits (i.e., *nad1*, *nad2*, *nad4*, *nad5* and *nad7)*, the *cox2* gene of the cytochrome c oxidase complex (CIV), the cytochrome c maturation gene *ccmFc*, as well as the ribosomal subunits *rpl2* and *rps3* (Best, et al. 2020a, b; Bonen 2008; Unseld, et al. 1997). We previously showed that *ABI3::PCIS1* plants exhibited defects in the processing of *nad4* and *nad7*, and presumably also in the maturation of *nad2* transcripts (Frink, et al. 2024). To further investigate the role of PCIS1 in mitochondrial gene expression and RNA metabolism, we analyzed the RNA profiles in embryo-rescued *pcis1-1* seedlings using RT-qPCR. The relative accumulation of mt-RNAs was determined by comparing rescued *pcis1-1* mutants with ‘embryo-germinated’ wild-type and heterozygous *pcis1* seedlings (i.e., green seeds collected from mature green siliques of heterozygous pcis1-1 plants and germinated on MS plates using the same procedure applied to rescue white homozygous *pcis1* seedlings), as well as with the ABI3::PCIS1 plantlet line. For this purpose, loci corresponding to all mitochondrial protein-coding genes, including their splice variants (exons flanking intron sequences), were analyzed by RT-qPCR using oligonucleotides designed to detect exon–exon or exon–intron junctions (Table S2). As shown in Fig. 2, comparison of transcript levels between homozygous *pcis1* mutants and wild type plants revealed notable reductions in several intron-containing RNAs. In particular, transcript levels for *nad4* exons 3–4 were reduced by 82.1- and 50.6-fold in *pcis1-1* and *pcis1-2*, respectively, while *nad7* exons 2–3 showed even greater reductions of 930.3- and 306.6-fold in *pcis1-1* and *pcis1-2*, respectively. The accumulation of transcripts corresponding to *nad2* exons 4–5 and *nad4* exons 1–2 and 2–3 was also affected, although to a lesser degree (i.e., approximately 1.5- to 3.5-fold lower in *pcis1-1* and *pcis1-2* than in wild type plants) (Fig. 2). The RT-qPCR analysis also revealed reductions in transcript levels corresponding to *nad4* exons 1–2 and exons 2–3 in both mutant lines, possibly resulting from processing defects affecting the maturation of *nad4* exons 3–4. As noted in other Arabidopsis mutants affected in mt-RNA metabolism (Best, et al. 2020a, b; Colas des Francs-Small and Small 2014; Small, et al. 2023), mutants maturation defects in *nad4*, *nad7* and plausibly in *nad2* were associated with small increases in the levels of many other mRNAs in the mutants. As also indicated previously (Frink, et al. 2024), the *ABI3::PCIS1* line exhibited reduced levels of *nad2* exons 4–5 (~ 16-fold decrease), *nad4* exon 3–4 (~ 42-fold decrease), and *nad7* exons 2–3 (~ 56-fold decrease) (Fig. S3).

RNA profiles in heterozygous (Htz; *pcis1-1*) and hemi-complemented *ABI3::PCIS1* plants were further analyzed and compared with wild type seedlings, all collected at the torpedo stage and germinated in vitro using the same procedure applied to rescue homozygous *pcis1* seedlings, to test and exclude potential transcriptional or post-transcriptional effects arising from the rescue procedure (Fig. S3). In contrast to *pcis1* mutants, mt-RNA processing, including transcripts from group II intron-containing genes, appeared unaffected in ‘embryo-germinated’ heterozygous (pcis1-1) plants (Fig. S3). These analyses indicate that PCIS1 is pivotal for the maturation of several mitochondrial transcripts in Arabidopsis, and that the observed defects in mt-RNA processing in *pcis1* are not attributable to the rescue procedure itself.

### PCIS1 functions in the splicing of *nad4* intron 3 and *nad7* intron 2 in Arabidopsis mitochondria

The processing defects we see in *nad4*, *nad7* and potentially *nad2* as well, likely indicate altered splicing in *pcis1* null mutants, as was previously suggested for the *ABI3::PCIS1* knockdown line (Frink, et al. 2024). To further establish the role(s) of PCIS1 in the processing of mitochondrial introns corresponding to *nad4*, *nad7*, and potentially *nad2* pre-RNAs, we compared the splicing efficiencies (i.e., the ratio of pre-RNAs to mRNAs) for the complete set of 23 group II-type introns in the Arabidopsis mitogenome (Unseld, et al. 1997), between embryo-germinated seedlings of wild type and *pcis1* mutant plants. Altered splicing activity was concluded only in cases where elevated levels of pre-RNAs in the mutant were accompanied by a reduction in transcripts containing the corresponding joined exons (i.e., mRNAs).

**Fig. 3:**
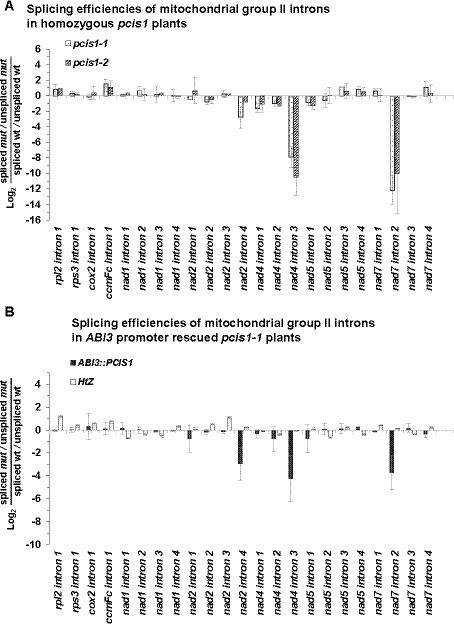
Reduced splicing efficiency of mitochondrial group II *nad4* intron 3 and *nad7* intron 2 in *pcis1* mutant plants. Total RNA extracted from whole seedlings of wild type (Col-0) and *pcis1-1* or *pcis1-2* mutant lines (panel A), as well as hemi-complemented *ABI3::PCIS1* and heterozygous *pcis1-1* (HtZ) plants (panel B), was analyzed by RT-qPCR. Transcript levels were normalized to reference genes, including *GAPDH* (*AT1G13440*), *ACTIN2* (*AT3G18780*), *18S rRNA* (*AT3G41768*), and mitochondrial 26S rRNA (*rrn26*, *ATMG00020*). Splicing efficiencies of the 23 mitochondrial group II introns were assessed as pre-RNA/mRNA ratios in mutant versus wild type plants. Histograms show the log2 fold change of relative RNA levels in *pcis1* lines compared with Col-0. Data represent the mean values of four biological replicates, each with 12 technical repeats; error bars indicate one standard deviation

The RT-qPCR data revealed large decreases in the processing (i.e., splicing efficiencies) of *nad4* intron 3 and *nad7* intron 2 in the *pcis1* mutants. Specifically, splicing efficiencies were reduced by approximately 240-fold and 1500-fold for *nad4* intron 3, and by ~4600-fold and ~1025-fold for *nad7* intron 2 in *pcis1-1* and *pcis1-2*, respectively, compared to wild-type seedlings (Fig. 3A). As previously shown (Frink, et al. 2024), the *ABI3::PCIS1* knockdown line exhibited reduced splicing efficiencies for *nad2* intron 4, *nad4* intron 3, and *nad7* intron 2, although to a lesser extent than in the homozygous *pcis1* mutants, specifically, showing 8-, 20-, and 13-fold reductions, respectively (Fig. 3B). The splicing efficiencies of the different intron RNAs in heterozygous *pcis1-1* mutant plants were comparable to those observed in wild type plants (Fig. 3B). However, we could not support a role for PCIS1 in the splicing of *nad2* intron 4, nor in the processing of *nad4* introns 1 and 2, although the corresponding mRNAs were found to be reduced in the *pcis1* mutants. We suspect that the decreased levels of *nad4* exon 1–2 and exon 2–3 in *pcis1-1* and *pcis1-2* are an indirect consequence of the splicing defects in the third intron within the *nad4* gene in the *pcis1* mutants. The altered processing of *nad2* transcripts may reflect a yet-unknown secondary effect. Similar effects on *nad2* pre-RNA processing were previously reported in various Arabidopsis mutants with impaired mt-RNA metabolism, potentially linked to broader changes in mitochondrial biogenesis and/or gene expression (Best, et al. 2023).

### The homozygous *pcis1* mutant plants show impaired biogenesis of the OXPHOS system

The altered mt-RNA metabolism observed in *pcis1* mutants is expected to affect mitochondrial function and overall plant physiology. The OXPHOS system comprises five core complexes: CI–CIV (electron transport chain) and CV (ATP synthase). In addition, plants possess bypass components, such as rotenone-insensitive NAD(P)H dehydrogenases (NDs) and alternative oxidases (AOXs) (Braun 2020; Møller, et al. 2021). The NAD2, and particularly the NAD4 and NAD7 subunits, are key components of mitochondrial CI and are essential for its proper biogenesis and function (Braun 2020; Klusch, et al. 2023). Accordingly, NAD4 is embedded within the membranous domain of CI and is believed to contribute to proton translocation across the inner mitochondrial (cristae) membrane, which is critical for establishing the proton motive force used in ATP biosynthesis. NAD7 is part of the peripheral (or matrix-exposed) arm and, although it likely does not play a direct role in electron transfer or proton pumping, it is found to be essential for the overall stability and proper function of complex I (Braun 2020; Ligas, et al. 2019; Møller, et al. 2021; Ostersetzer-Biran 2016).

**Fig. 4:**
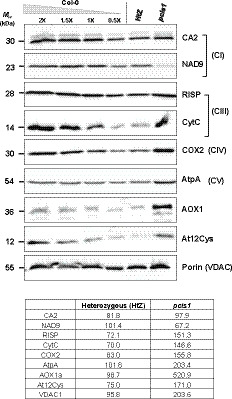
Accumulation of various organellar proteins in wild type and *pcis1* plants. Crude mitochondrial (and plastidial) membrane fractions were isolated from wild type (Col-0) and *pcis1-1* mutant plants. Equal amounts of protein were separated by SDS-PAGE and analyzed by immunoblotting with specific antibodies raised against representative organellar proteins. Signals were detected using chemiluminescence (ECL) and recorded on an imaging system (ImageQuant LAS 4000, GE Healthcare, Haifa, Israel)

Western blot analyses of MS-grown wild type and *pcis1* mutant plants revealed that the steady-state levels of CI subunits CA2 (~ 30 kDa; showed comparable levels to that in wild type plants) were not strongly affected by the loss of PCIS1 activity, while NAD9 (~ 23 kDa) was reduced by about twofold in *pcis1* (Fig. 4). Several subunits from other organellar complexes (e.g., RISP or CytC of CIII, Cox2 of CIV, AtpA of CV, and the outer membrane VDAC1 protein) were found to accumulate to higher levels in homozygous *pcis1* plants. Notably, AOX1a (~ 35 kDa) accumulated to ~ 50-fold higher levels, consistent with its upregulation in plants affected in the OXPHOS and under various stress conditions (Møller, et al. 2021). Likewise, a marked increase in the accumulation of the At12Cys protein, which is known to be upregulated in CI-deficient cells (Wang, et al. 2016), was observed in the *pcis1-1* homozygous line (Fig. 4). Protein levels in the heterozygous *pcis1-1* plants were comparable to those in wild-type plants, although modest reductions in the steady-state levels of several proteins, including CA2, RISP, CytC, and At12Cys, were apparent (Fig. 4).

**Fig. 5:**
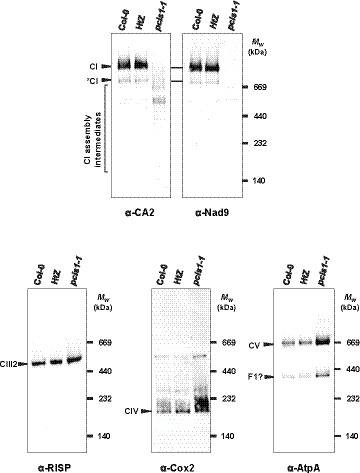
The *pcis1* mutants display altered biogenesis of the respiratory complex I. The accumulation of respiratory complexes in wild type (Col-0), heterozygous (*PCIS1/pcis1-1*) and homozygous *pcis1-1* plants was analyzed by BN-PAGE on crude organellar membrane preparations (Pineau, et al. 2008). Following electrophoresis, proteins were transferred from the native gels onto PVDF membranes and analyzed by immunoblotting with the indicated antibodies. Signal detection was carried out using chemiluminescence. Arrows mark the positions of the native complexes: complex I (CI, ~ 1,000 kDa), complex III dimer (CIII₂, ~ 500 kDa), complex IV (CIV, ~ 220 kDa), and complex V (CV, ~ 660 kDa). An asterisk in the CA2 panel indicates the last assembly intermediate (∼850 kDa band) in the CI assembly pathway (CI*), while lower molecular mass particles indicate CI-assembly intermediates

In line with the RNA profiles and the steady-state levels of various organellar proteins, BN-PAGE followed by immunoblot analyses, confirmed a strong reduction (below detectable levels) of native CI in the homozygous *pcis1* plants (Fig. 5). We also noticed several lower molecular weight CA2-containing particles in the blot, likely indicating the presence of CI membrane-arm assembly intermediates, as also described for various CI-deficient Arabidopsis mutants (Colas des Francs-Small and Small 2014; Ligas, et al. 2019). No detectable holo-CI, nor CI-containing supercomplexes, were observed using the soluble arm anti-NAD9 subunit, while typical CI-related particles were observed in heterozygous *pcis1* and the embryo-germinated wild-type (Col-0) seedlings. Higher CIII dimmers, CIV and CV levels were seen in *pcis1*, along with a ~ 400 kDa cluster in the AtpA blot, plausibly reflecting the ATP-synthase F1 module (Chen, et al. 2024; Senkler, et al. 2017), which was also upregulated in the homozygous mutant plants.

### Arabidopsis *pcis1* mutants display complex I-specific respiratory defects

**Fig. 6:**
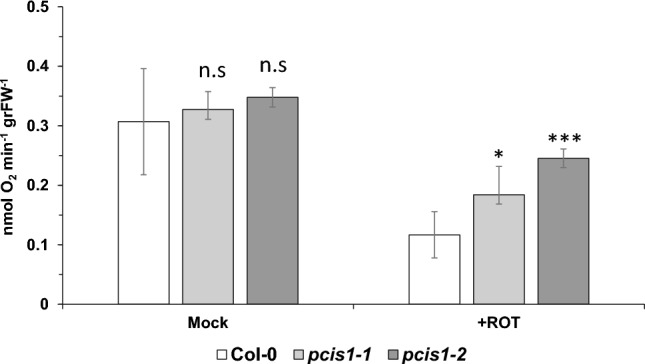
Impaired respiratory complex I activity in *pcis1* mutants. Mitochondrial respiratory activities were measured in wild type and *pcis1* mutant plants by monitoring oxygen uptake rates in the dark. Oxygen consumption (i.e., O_2_-uptake rates) was assayed in intact seedlings using a Clark-type oxygen electrode. Rotenone CI inhibitor treatments were applied to dissect the contribution of respiration through CI. Compared with wild type, *pcis1* mutants exhibited markedly reduced complex I-dependent respiration, consistent with defects in NADH oxidation. Based on the respiratory data we speculate that oxygen uptake was largely maintained by alternative dehydrogenases (NDs) and/or the alternative oxidase (AOX) pathway, as indicated by the upregulation in AOXs in Fig. 4. Error bars represent ± SD of biological replicates (*n* ≥ 5). Asterisks indicate significant differences [i.e., *P ≤ 0.05, **P ≤ 0.01, ***P ≤ 0.001, or not significant (ns)] between the wild type and mutant plants

The RNA and protein profiles indicate altered respiratory functions in *pcis1* mutants. The oxygen-uptake rates of excised tissues from wild-type plants and *pcis1* mutant seedlings were measured in the dark (Fig. 6). The average O_2_-uptake rates of *pcis1-1* and *pcis1-2* (0.33 ± 0.09 and 0.35 ± 0.02 nmol O_2_ min^−1^ grFW^−1^, respectively) were equivalent to that of the 14-day-old Col-0 seedlings (0.31 ± 0.09 nmol O_2_ min^−1^ grFW^−1^). The respiratory functions in wild type and *pcis1* mutants were further analyzed in the presence of the electron transport inhibitor rotenone (ROT), a specific complex I inhibitor. Pre-incubation with rotenone (50 µM) had a strong effect on the respiration rates of wild type plants (i.e., 0.12 ± 0.04 nmol O_2_ min^−1^ gr FW^−1^), about 61% lower than the respiration activity under standard conditions (Fig. 6, Mock). As also seen in various other mutants affected in CI biogenesis or activity, rotenone had a milder effect (i.e., between 29 ~ 45% inhibition) on the respiratory activities of *pcis1* mutant plants (*i.e*. 0.18 ± 0.03 and 0.25 ± 0.02 nmol O_2_ min^−1^ grFW^−1^) (Fig. 6). These data further indicate that CI was strongly affected in *pcis1* mutants, and that the observed O_2_-uptake rates in the mutant likely coincide with the upregulation of alternative pathways of electron transport, via rotenone-insensitive type II NAD(P)H dehydrogenases (NDs) and alternative oxidases (AOXs, Fig. 4), which can bypass CI and CIV, respectively.

## Discussion

In this study, we established homozygous *pcis1* mutant lines using modified embryo rescue approaches, enabling an *in-depth* functional analysis of PCIS1, a mitochondrially localized protein essential for Arabidopsis embryogenesis (Frink, et al. 2024). Our genetic, molecular and biochemical analyses demonstrate that PCIS1 plays a pivotal role in the maturation and processing of *nad4* intron 3 and *nad7* intron 2. In contrast, the altered levels of *nad2* transcripts (Figs. 2, 3, S3) likely represent a secondary effect of mitochondrial dysfunction, as also noted previously (Best, et al. 2023). These findings expand upon earlier work on *ABI3::PCIS1* knockdown plants (Frink, et al. 2024) by revealing the specific molecular and physiological consequences of complete PCIS1 loss-of-function on the biogenesis of the mitochondrial respiratory system.

### Link between PCIS1-dependent splicing, complex I biogenesis, and respiratory functions

The mt-RNA processing defects observed in null *pcis1* mutants indicate notable reductions in the splicing efficiencies of *nad4* intron 3 and *nad7* intron1 1 and 2 transcripts, and appear to indirectly affect the processing of *nad2* pre-mRNAs as well (Figs. 2, 3, S3). Notably, the magnitude of the mt-RNA processing defects in the homozygous null mutants (240-fold and 1500-fold for *nad4* intron 3, and by ~ 4600-fold and ~ 1025-fold for *nad7* intron 2 in *pcis1-1* and *pcis1-2*, respectively) (Fig. 3A), exceeded those of *ABI3::PCIS1* plants (i.e., 19.2-fold and 13.2-fold for *nad4* intron 3 and *nad7* intron 2, respectively) (Frink, et al. 2024) (Fig. 3B and S3), highlighting the effects of residual PCIS1 activities in partially sustaining mt-RNA metabolism.

The correct maturation of these transcripts is critical for the expression of the corresponding NAD4, NAD7, and NAD2 subunits of complex I (CI), and consequently for the assembly of a functional holo-CI and CI-containing respirasomal supercomplexes (Braun, et al. 2014; Meyer 2012; Meyer, et al. 2011, 2019; Ostersetzer-Biran 2016) in plant mitochondria. Loss of either subunit destabilizes the assembly and activity of the CI holoenzyme in Arabidopsis, and thus prevents its incorporation into respiratory supercomplexes, thereby impairing mitochondrial electron transport; see e.g., (Braun, et al. 2014; Ligas, et al. 2019; Meyer, et al. 2011; Ostersetzer-Biran 2016). Consistent with this, *pcis1* mutants exhibit a major reduction of CI (Fig. 5), which is accompanied by the appearance and accumulation of several CI assembly intermediates (Fig. 5) and upregulation of other OXPHOS enzymes (Fig. 5). These include AtCys12 and AOX1/2 proteins (Fig. 4) and, presumably alternative NDs, which are known to be co-upregulated with AOXs under stress and altered respiratory conditions (Clifton, et al. 2005). The *pcis1* mutant phenotypes, and the reduced sensitivity of *pcis1* mutants’ respiration rates to rotenone (Fig. 6, + ROT), mirror those seen in other CI-deficient Arabidopsis mutants (Braun, et al. 2014; Kuhn, et al. 2015; Ligas, et al. 2019; Møller, et al. 2021; Ostersetzer-Biran 2016). Despite the loss of CI activity, overall mitochondrial respiration was apparently not significantly reduced (or may even slightly upregulated) (Fig. 6). These combined activities of alternative respiratory enzymes (e.g., AOXs and rotenone-insensitive NDs), together with other OXPHOS complexes (e.g., CIII, CIV, and CV) that are upregulated in the mutants, may provide bypass routes for electron flow through the respiratory chain and partially compensate for the impaired CI-associated electron transport (Braun 2020; Braun, et al. 2014; Garmier, et al. 2008; Kuhn, et al. 2015; Møller, et al. 2021; Rasmusson and Møller 2015; Schertl and Braun 2014), thereby assisting the cells in maintaining mitochondrial redox balance and sustaining respiration rates under the altered physiological conditions of the mutants.

The absence of comparable effects in heterozygous mutant lines further underscores the recessive nature of the *pcis1* mutations and may indicate that CI assembly and activity are not strongly compromised when PCIS1 levels are reduced but still sufficient.

### Developmental and physiological consequences

The embryogenesis defects in *pcis1* mutants (Fig. 1), arrested at the torpedo stage, retarded seed germination and early seedling lethality under in vivo conditions, are characteristic of mitochondrial gene expression mutants with severe CI defects (Braun, et al. 2014; Colas des Francs-Small and Small 2014; Kuhn, et al. 2015; Ligas, et al. 2019; Møller, et al. 2021; Ostersetzer-Biran 2016). Given the central role of CI in generating the proton motive force for ATP synthesis, and presumably also for ascorbate biosynthesis (Bartoli, et al. 2000; Schertl, et al. 2012; Schimmeyer, et al. 2015), the PCIS1-mediated splicing defects likely compromise mitochondrial bioenergetics, particularly during high energy-demanding stages, such as embryo development, seed germination, and early seedling establishment (Kimata, et al. 2020; Nietzel, et al. 2020; Ostersetzer-Biran 2016). The induction of some stress-responsive mitochondrial proteins in *pcis1* plants (Fig. 4) further supports a model in which PCIS1 loss triggers broad metabolic reprogramming required to mitigate the effects of impaired OXPHOS functions (Best, et al. 2020a, b; Kuhn, et al. 2015; Ostersetzer-Biran 2016). Altered respiratory functions can limit the energy available for biosynthetic processes, while altered redox balance and metabolic fluxes disrupt e.g., cellular signaling, nutrient mobilization, or stress adaptation. These combined effects result in altered embryogenesis, delayed germination, and impaired seedling establishment, underscoring the essential role of mitochondria in supporting energy-intensive stages of plant development.

### Distinct physiological outcomes of PCIS1 ‘downregulation’ versus null mutation in seed plants

By analyzing null *pcis1* and ‘hemi-complemented’ *ABI3::PCIS1* (i.e., a developmentally restricted complementation line, functionally resembling a knockdown outside developmental window of embryogenesis and early seedling establishment) mutant plants, our study highlights the functional threshold of PCIS1 in mt-RNA metabolism. While the different genotypes show equivalent defects in the processing of introns residing in *nad4* and *nad7*, the residual PCIS1 activity in the conditionally complemented (and heterozygous plants) likely decreases the severity of the mt-RNA processing defects and thus maintains a basal CI activity sufficient for cellular activities and for seedling growth and development. The impaired maturation of *nad2* pre-RNA transcripts observed in *ABI3::PCIS1* mutant plants may reflect an indirect effect of an as-yet-unknown mechanism in organellar gene expression, as *nad2* processing is less affected in homozygous *pcis1* mutants (Figs. 2, 3, S3). Accumulation of mitochondrial pre-RNAs may act as a sink (i.e., sequestration/titration effect) for factors required for *nad2* processing, thus affecting splicing efficiency in the mutant lines (Best, et al. 2023). We further noted that complete loss of PCIS1 in homozygous lines profoundly disrupts the maturation of *nad4* and *nad7* transcripts, thereby impairing holo-CI biogenesis and activity (Figs. 5, 6), resulting in embryonic developmental arrest (Figs. 1, S2). Such differences between different mutant lines underscore the need for detailed interpretation of knockdown-like phenotypes, particularly when studying the functions of essential genes in organellar biogenesis and function, and highlight the importance of integrating genetic, molecular, and biochemical analyses of both null mutants and downregulated lines to fully elucidate gene function.

Taken together, our findings establish PCIS1 as an essential factor for the splicing of a subset of mitochondrial group II introns, linking its activity to CI biogenesis and respiratory functions, and hence to embryonic development, seed germination and development in Arabidopsis. The specificity of the splicing defects suggests that PCIS1 either acts as a processing cofactor or promotes the assembly of intron-specific ribonucleoprotein splicing complexes for three intron pre-RNA substrates. Ongoing studies aim to define its molecular interactions within the mitochondrial splicing machinery and determine whether PCIS1 homologs in other plant species exhibit similar target specificity.

## Supplementary Information

Below is the link to the electronic supplementary material.

Figure S1. Expression patterns of PCIS1 across tissues and developmental stages. Expression levels and patterns of PCIS1 were analyzed using the publicly available ‘The Arabidopsis Information Resource’ website (TAIR; http://www.arabidopsis.org) (Reiser, et al. 2022). Supplementary file1 (EPS 2143 KB)

Figure S2. Germination and developmental phenotypes associated with pcis1 mutants. Seeds collected from mature siliques of wild type plants and heterozygous pcis1 mutants (A) were germinated on MS-agar plants supplemented with 1% sucrose (B). (C) 3-month-old homozygous pcis1-1 mutant seedlings grown under long-day conditions (18 h light/6 h dark; ~100 µE·m⁻²·s⁻¹, 50% RH, 22°C). (D) Mature green siliques of homozygous pcis1-1 mutants. Scale bars are shown below each panel. Supplementary file2 (EPS 21182 KB)

Figure S3. Accumulation of mitochondrial protein-coding transcripts in ABI3::PCIS1 and heterozygous pcis1-1 mutant plants. Total RNA extracted from whole seedlings of wild type (Col-0) plants and pcis1 (i.e., ABI3::PCIS1 and heterozygous pcis1-1) mutant lines, was analyzed by reverse transcription followed by quantitative real-time PCR (RT-qPCR). Transcript levels were normalized to multiple internal references, including GAPDH (AT1G13440), ACTIN2 (AT3G18780), 18S rRNA (AT3G41768), and rrn26 (mitochondrial 26S rRNA, ATMG00020). RT-qPCR was performed to assess the expression profiles of mRNAs corresponding to the 32 protein-coding genes in Arabidopsis mitochondria. The histograms show the log2 ratio of the relative RNA levels in rpd1 lines compared with those of the wild type plants. Data represent the mean values of four biological replicates, each with 12 technical repeats; error bars indicate one standard deviation. Supplementary file3 (EPS 858 KB)

Supplementary file4 (PDF 115 KB)

Supplementary file5 (PDF 182 KB)

Supplementary file6 (PDF 121 KB)

## Data Availability

All data supporting this study are included in the article and supplementary information; additional data are available from the corresponding author (oren.ostersetzer@mail.huji.ac.il) upon reasonable request.
