# Supplementary material for: PCIS1 is an essential factor in mitochondrial RNA splicing and complex I biogenesis, with distinct effects in null and downregulated mutants
Source: Plant Cell Rep. 2026 Jun 29;45(7):210. doi: 10.1007/s00299-026-03821-w (PMC13315167; doi:10.1007/s00299-026-03821-w)
Supplement: Supplementary file 4 — Supplementary file4 (PDF 115 KB) [file 299_2026_3821_MOESM4_ESM.pdf]

**Table S1.** List of oligonucleotides used for the screening of *pcis1* mutants in this study.

| Gene / Construct | Forward oligo (5’→3’) | Reverse oligo (5’→3’) |
|---|---|---|
| *PCIS1.1 (At5g25500)* | CCCAATTTCGCCGTTTCTTCA | GCGTTACCTGTGAACACACA |
| *PCIS1.2 (At5g25500)* | CCCAATTTCGCCGTTTCTTCA | ACAGGTAGTTGCTTGTACCTCA |
| *ABI3::PCIS1* | see Frink et al. (2024) Plant Cell Physiol, 65, 1474-1485 (doi: 10.1093/pcp/pcae086) | |
| *LB1.3 (T-DNA)* | --- | ATTTTGCCGATTTCGGAAC |
