# Supplementary material for: PCIS1 is an essential factor in mitochondrial RNA splicing and complex I biogenesis, with distinct effects in null and downregulated mutants
Source: Plant Cell Rep. 2026 Jun 29;45(7):210. doi: 10.1007/s00299-026-03821-w (PMC13315167; doi:10.1007/s00299-026-03821-w)
Supplement: Supplementary file 5 — Supplementary file5 (PDF 182 KB) [file 299_2026_3821_MOESM5_ESM.pdf]

Table S2. List of RT-qPCR oligonucleotides designed to specific different exon-exon (analysis of mRNA and processed transcripts) and intron-exon regions (analysis of pre-RNAs) in Arabidopsis mitochondria.

a. List of oligonucleotides used for the analysis of the mRNA profiles in wild type and mutant plants

gene target	oligo's name	sequence (5'→3')
atp1	*atp1F*	TCACTTCGACACGTCTTTGC
	atp1R	GGAATGGCCTTGAATCTTGA
atp6	*atp6-1F*	TCTTTTGCGAGTCAATGCAC
	atp6-1R	TCTCGCGTATCTCACATTGC
atp8	*atp8F*	CCGTCGACTTATTGGGAAAA
	atp8R	TTCCTTGGCCATGTACAACA
atp9	*atp9F*	CATTCCCTCTGACGTCGAAT
	atp9R	TCGTCGATTCTTACCCTCGT
atp4	*atp4F*	GGATCAGCTTGCGAATTTGT
	atp4R	GCAAATTGCTTCCCCACTAA
ccmb	*ccmBF*	TCTTGGAATCACATCCAGCA
	ccmBR	CGAGACCGAAATTGGAAAAA
ccmc	*ccmCF*	AGCTACGCGCAAATTCTCAT
	ccmCR	GCCGTGGCGATATAAACAAT
ccmfc	*ccmFcF*	CACATGGAGGAGTGTGCATC
	ccmFcR	GTGGGTCCATGTAAATGATCG
ccmfn-1	*ccmFN1F*	AGCTCTTGGCATTGCTTTGT
	ccmFN1R	AGTGCCACAATCCCATTCAT
ccmfn-2	*ccmFN2F*	CGTGTCGTTCGTAATGGAAA
	ccmFN2R	TGATAAGCCCACCAACTTCC
cob	*cobF*	TGCCGGAATGGTATTTCCTA
	cobR	GCCAAAAGCAACCAAAACAT
cox1	*cox1F*	GTAGCTGCGGTGAAGTAGGC
	cox1R	CTGCCTGGATTCGGTATCAT
cox2	*cox2F*	TGATGCTGTACCTGGTCGTT
	cox2R	TGGGGGATTAATTGATTGGA
cox3	*cox3F*	CCGTAACTTGGGCTCATCAT
	cox3R	AAACCATGAAAGCCTGTTGC
mttb	*mttBF*	GGGGTCTTTCTTTGGAAACC
	mttBR	TCTCCCTCATTCCACTCGTC
nad1 exons 1-2	*nad1 1-2F*	GACCAATAGATACTTCATAAGAGACCA
	nad1 1-2R	TTGCCATATCTTCGCTAGGTG
nad1 exons 2-3	*nad1 2-3F*	ATTCAGCTTCCGCTTCTGG
	nad1 2-3R	TCTGCAGCTCAAATGGTCTC
nad1 exons 3-4	*nad1 3-4F*	AAAAGAGCAGACCCCATTGA
	nad1 3-4R	TCCGTTTGATCTCCCAGAAG
nad1 exons 4-5	*nad1 4-5F*	AGCCCGGGATCTTCTTGA
	nad1 4-5R	TCTTCAATGGGGTCTGCTC
nad2 exons 1-2	*nad2 exons 1-2F*	GCGAGCAGAAGCAAGGTTAT
	nad2 exons 1-2R	GGATCCTCCCACACATGTTC
nad2 exons 2-3	*nad2 exons 2-3F*	AAAGGAACTGCAGTGATCTTGA
	nad2 exons 2-3R	AATATTTGATCTTAGGTGCATTTTC
nad2 exons 3-4	*nad2exons 3-4F*	GCGCAATAGAAAGGAATGCT
	nad2exons 3-4R	CTATGGGTCTACTGGAGCTACCC
nad2 exons 4-5	*nad2exons 4-5F*	CAAAGGAGAGGGGTATAGCAA
	nad2exons 4-5R	TATTTGTTCTTCGCCGCTTT
nad3	*nad3F*	CGAATGTGGTTTCGATCCTT
	nad3R	GCACCCCTTTTCCATTCATA
nad4 exons 1-2	*nad4 exons 1-2F*	ATTCTATGTTTTTCCCGAAAGC
	nad4 exons 1-2R	GAAAAACTGATATGCTGCCTTG
nad4 exons 2-3	*nad4 exons 2-3F*	AATACCCATGTTTCCCGAAG
	nad4 exons 2-3R	TGCTACCTCCAATTCCCTGT

nad4exons 3-4	*nad4exons 3-4F*	TTCCTCCATAAATTCTCCGATT
	nad4exons 3-4R	TGAAATTTGCCATGTTGCAC
nad4L	*nad4L-F*	GGGGAATCCTCCTTAATAGACG
	nad4L-R	AACGAAAATGGCTAACCCAATA
nad5 exons 1-2	*nad5 exons 1-2F*	TGGACCAAGCTACTTATGGATG
	nad5 exons 1-2R	CCATGGATCTCATCGGAAAT
nad5 exons 2-3	*nad5 exons 2-3F*	TACCTAAACCAATCATCATATC
	nad5 exons 2-3R	CTGGCTCTCGGGAGTCTCTT
nad5 exons 3-4	*nad5exons 3-4F*	AACTCGGATTCGGCAAGAA
	nad5exons 3-4R	GATATGATGATTGGTTTAGGTA
nad5 exons 4-5	*nad5exons 4-5F*	AACATTGCAAAGGCATAATGA
	nad5exons 4-5R	GTTCCTGCGTTTCGGATATG
nad6	*nad6F*	TATGCCGGAAAGGTACGAAG
	nad6R	GTGAGTGGGTCAGTCGTCCT
nad7 exons 1-2	*nad7 exons 1-2F*	ACCTCAACATCCTGCTGCTC
	nad7 exons 1-2R	AAGGTAAAGCTTGAAGATAAGTTTTGT
nad7 exons 2-3	*nad7 exons 2-3F*	GAGGGACTGAGAAATTAATAGAGTACA
	nad7 exons 2-3R	TGGTACCTCGCAATTCAAAA
nad7 exons 3-4	*nad7exons 3-4F*	ACTGTCACTGCACAGCAAGC
	nad7exons 3-4R	CATTGCACAATGATCCGAAG
nad7 exons 4-5	*nad7exons 4-5F*	GATCAAAGCCGATGATCGTAA
	nad7exons 4-5R	AGGTGCTTCAACTGCGGTAT
nad9	*nad9F*	GGATGACCCTCGAAACCATA
	nad9R	CACGCATTCGTGTACAAACC
rpl2	*rpl2F*	CCGAAGACGGATCAAGGTAA
	rpl2R	CGCAATTCATCACCATTTTG
rpl5	*rpl5F*	AAGGGGTTCGACAGGAAAGT
	rpl5R	CGTATTTCGACCGGAAAATC
rpl16	*rpl16F*	GAGCATTTGCCAAACTCACA
	rpl16R	CGGACACTTTCATCGTGCTA
rps3	*rps3F*	CCGATTTCGGTAAGACTTGG
	rps3R	AGCCGAAGGTGAGTCTCGTA
rps4	*rps4F*	ACCCATCACAGAGATGCACA
	rps4R	TCACACAAACCCTTCGATGA
rps7	*rps7F*	CTCGAACTGAACGCGATGTA
	rps7R	AAGCTGCTTCAAGGATCCAA
rps12	*rps12F*	AGCCAAAGTACGGTTGAGCA
	rps12R	TTTGGGTTTTTCTGCACCAT
matR	*matR-F*	AATTTTTGCGAGAGCTGGAA
	matR-R	TTGAACCCCGTCCTGTAGAC
rrn18	*rrn18F*	CGTCACCTGGGTCAAAAACT
	rrn18R	GCTTGAAAACCGAAGTGAGC
rrn26	*rrn26F*	GACGAGACTTTCGCCTTTTG
	rrn26R	CTTGGAGCGAATTGGATGAT
rrn5	*rrn5F*	CCGACCTCGATATGTGGAATCGTC
	rrn5R	TGGACCATGTCTCCCGAACAATC
18S rRNA (At3g41768)	*18S nucl-F*	AAACGGCTACCACATCCAAG
	18S nucl-R	ACTCGAAAGAGCCCGGTATT
ACTIN2 (At3g18780)	*actin2-F*	GGTAACATTGTGCTCAGTGGTGG
	actin2-R	AACGACCTTAATCTTCATGCTGC
GAPDH (At3g04120)	*GAPDH-F*	TCTCGATCTCAATTTCGCAAAA
	GAPDH-R	CGAAACCGTTGATTCCGATTC

b. List of oligonucleotides designed for the analysis of the 23 organellar group II introns in Arabidopsis.

**Gene**	**Forward oligo (5'→3')**	**Reverse oligo (5'→3')**
rpl2 intron 1 exon 2	TTAGGAAGAGCCGTACGAGG	CGCAATTCATCACCATTTTG
rps3 intron 1 exon 2	AGCCGAAGGTGAGTCTCGTA	TCTACGGCGGGGTCACTAT

cox2 intron 1 exon 2	TGGGGGATTAATTGATTGGA	AGCAGTACGAGCTGAAAGGC
ccmFc intron 1 exon 1	CCCGGATCGAATCAGAGTT	CACATGGAGGAGTGTGCATC
nad1 intron 1 exon 2	GACCAATAGATACTTCATAAGAGACCA	CGTGCTCGTACGGTTCATAG
nad1 intron 2 exon 2	GGTTGGGTTAGGGGAACATC	TCTGCAGCTCAAATGGTCTC
nad1 intron 3 exon 4	AAAAGAGCAGACCCCATTGA	GGGAGCTGTATGAGCGGTAA
nad1 intron 4 exon 5	AGCCCGGGATCTTCTTGA	ACGGAGCTGCATCCCTACT
nad2 intron 1 exon 2	GCGAGCAGAAGCAAGGTTAT	CCCATTCCTAACCAGTGGAG
nad2 intron 2 exon 2	CCCGATCCGATAGTTTACAA	AATATTTGATCTTAGGTGCATTTTC
nad2 intron 3 exon 4	GCGCAATAGAAAGGAATGCT	GGCGAATTTCAAACTTGTGG
nad2 intron 4exon 4	CTTATTCGTGGCAACCTTCC	TATTTGTTCTTCGCCGCTTT
nad4 intron 1 exon 2	CCGTATGATGCGGAAGTCTC	GAAAAACTGATATGCTGCCTTG
nad4 intron 2 exon 3	GCGGAACGACCAGAAAAATA	TGCTACCTCCAATTCCCTGT
nad4 intron 3 exon 4	TCTAGCTTGGTTCGGAGAGC	TGAAATTTGCCATGTTGCAC
nad5 intron 1 exon 2	TGGACCAAGCTACTTATGGATG	TTCGCAAATAGGTCCGACT
nad5 intron 2-exon 2	GTACGATCGTGTCGGGTGA	CTGGCTCTCGGGAGTCTCTT
nad5 intron 3-exon 4	AACTCGGATTCGGCAAGAA	GCCGTGTAATAGGCGACCA
nad5 intron 4 exon 5	AACATTGCAAAGGCATAATGA	CCTGTAAACCCCCATGATGT
nad7 intron 1 exon 2	ACGGTTTTTAGGGGGATCTG	AAGGTAAAGCTTGAAGATAAGTTTTGT
nad7 intron 2 exon 3	AGTGGGAGAGCCGTGTTATG	TGGTACCTCGCAATTCAAAA
nad7 intron 3 exon 4	TAAAGTGAAGTGGTGGGCCT	CATTGCACAATGATCCGAAG
nad7 intron 4 exon 5	CGGCCAAATGACTACAGGAT	AGGTGCTTCAACTGCGGTAT
