# Supplementary material for: PCIS1 is an essential factor in mitochondrial RNA splicing and complex I biogenesis, with distinct effects in null and downregulated mutants
Source: Plant Cell Rep. 2026 Jun 29;45(7):210. doi: 10.1007/s00299-026-03821-w (PMC13315167; doi:10.1007/s00299-026-03821-w)
Supplement: Supplementary file 6 — Supplementary file6 (PDF 121 KB) [file 299_2026_3821_MOESM6_ESM.pdf]

Table S3. List of antibodies used for the analysis of organellar proteins in wild type and *pcis1* mutant plants.

Antibody	Protein I.D.	origin	serum	dilution	Reference / source
At12Cys	CHCH domain protein	*Arabidopsis thaliana*	Rabbit (polyclonal)	1/1,000	[3]
AOX1a	Ubiquinol (Alternative) oxidase 1a	*Arabidopsis thaliana*	Rabbit (polyclonal)	1/1,000	Agrisera antibodies, AS04 054
AtpA	Mitochondrial ATP-synthase subunit 1 (A)	*Nicotiana tabacum*	Mouse (monoclonal)	1/500	Thomas Elton collection
CA2	g-carbonic anhydrase-like subunit 2	*Arabidopsis thaliana*	Rabbit (polyclonal)	1/1,000	[1],[2]
COX2	Cytochrome C oxidase subunit-2	*Nicotiana tabacum*	Mouse (monoclonal)	1/100	Thomas Elton collection
CytC	Cytochrome C	*Arabidopsis thaliana*	Rabbit (polyclonal)	1/1,000	Agrisera antibodies, AS08 343A
NAD9	NADH-dehydrogenase complex subunit-9	*Triticum spp.*	Rabbit (polyclonal)	1/50,000	(Lamattina et al., 1993)
RISP	Rieske iron-sulfur protein	*Arabidopsis thaliana*	Rabbit (polyclonal)	1/5,000	Gift of Prof. Ian Small, UWA
Porin/VDAC	Voltage-dependent anion-selective channel protein 1-5	*Arabidopsis thaliana*	Rabbit (polyclonal)	1/1,000	Agrisera antibodies, AS07 212

1. Perales M, Eubel H, Heinemeyer J, Colaneri A, Zabaleta E, Braun H-P: Disruption of a nuclear gene encoding a mitochondrial gamma carbonic anhydrase reduces complex I and supercomplex I+III2 levels and alters mitochondrial physiology in Arabidopsis. J Mol Biol 350: 263-277 (2005).
2. Sunderhaus S, Dudkina NV, Jansch L, Klodmann J, Heinemeyer J, Perales M, Zabaleta E, Boekema EJ, Braun HP: Carbonic anhydrase subunits form a matrix-exposed domain attached to the membrane arm of mitochondrial complex I in plants. J Biol Chem 281: 6482-6488 (2006).
3. Wang Y, Lyu W, Berkowitz O, Radomiljac JD, Law SR, Murcha MW, Carrie C, Teixeira PF, Kmiec B, Duncan O, Van Aken O, Narsai R, Glaser E, Huang S, Roessner U, Millar AH, Whelan J: Inactivation of mitochondrial complex i induces the expression of a twin cysteine protein that targets and affects cytosolic, chloroplastidic and mitochondrial function. Mol Plant 9: 696-710 (2016).
